# Knowledge and management practices of infant teething symptoms among mothers in a tertiary facility in Ghana

**DOI:** 10.11604/pamj.2024.47.65.40792

**Published:** 2024-02-13

**Authors:** Abubakari Wuni, Mohammed Iddrisu, Aloysius Ali Angliengmene, Solomon Mohammed Salia, Letitia Chanayireh, Iddrisu Sisala Mohammed, Ajara Musah, Mudasir Mohammed Ibrahim, Anthoinette Afua Kpentey, Cecilia Kwarteng, Brenda Abena Nyarko, Abdul Razak Doat

**Affiliations:** 1 Department of Medicine for the Elderly (C6), Cambridge University Hospitals National Health Service Foundation Addenbrookes Hospital, Cambridge, United Kingdom,; 2 Nurses and Midwives Training College, Tamale, Ghana,; 3 Department of Nursing, School of Nursing and Midwifery, University of Health and Allied Sciences, Ho, Ghana,; 4 Department of Midwifery and Women´s Health, School of Nursing and Midwifery, University for Development Studies, Tamale, Ghana,; 5 Regentropfen College of Applied Sciences, Private Mail Bag, Bolgatanga, Upper East Region, Ghana,; 6 School of Nursing and Midwifery, Clement Kubindiwor Tedam University of Technology and Applied Sciences Navrongo, Upper East, Ghana

**Keywords:** Infant, teething, knowledge, practice, mothers

## Abstract

**Introduction:**

teething is a natural process that all infants go through, and most toddlers obtain their first tooth around six months. However, misconceptions about teething and its remedies are still prevalent. The study assessed the knowledge and management practices of infant teething symptoms among mothers whose children were admitted to the Pediatric ward of Tamale Teaching Hospital.

**Methods:**

the study adopted a prospective descriptive cross-sectional design with a quantitative data collection method. A total of 251 mothers were selected using a convenient sampling strategy, and a structured questionnaire was used for data collection.

**Results:**

the study found that 79.7% and 20.3% of respondents had good and poor knowledge of teething, respectively. Also, 65.3% and 34.7% of the mothers had good and poor practices, respectively, in the management of teething symptoms. Marital status (p= 0.029) and type of ward (p= 0.020) were significantly associated with mothers' knowledge of teething. Furthermore, mothers less than 30 years of age (OR, 2.07; 95% CI: 1.19-3.57; p= 0.009) and mothers with formal education (OR, 2.22; 95% CI: 1.22-3.81; p= 0.004) were more likely to have good management practices for teething symptoms.

**Conclusion:**

most mothers have a good understanding of child teething, but they do not think delayed eruptions indicate systemic disease. They identified taking the child to the hospital during teething symptoms and administering Paracetamol to relieve the symptoms as standard practices. However, more education is needed to differentiate between teething signs and other ailments and to prevent substandard first aid practices during teething.

## Introduction

Every child, once born, is bound to go through a developmental milestone (normal or abnormal) to adjust to the ecological changes in the environment. Infants undergo physical, emotional, physiological, and social development. Physical development is objective and easily noticeable to oneself and others. One aspect of physical development with physiological implications is teething. Teething is a natural process that all infants experience, and most toddlers develop their first tooth around six months [1,2], and have a complete set of 20 deciduous teeth by 30 months of age [1]. Parental perceptions and beliefs about teething vary, often influenced by the symptoms a child experiences during tooth eruption. Individuals from diverse backgrounds, spanning education levels, medical knowledge, gender, rural or urban residency, wealth, and age, associate different symptoms with tooth eruption or teething. Tsang [3] contends that, despite teething being a normal part of infant development, there is surprisingly little known about the causes and management of teething signs and symptoms. Teething is seen as a discomforting process for the child [4]. Manifestations of teething include agitation, bowel upset (including diarrhea, constipation, loose stools), colic, convulsions, cough, croup, ear rubbing/pulling, excessive saliva and drooling, facial flushing, fever, inflamed/irritated gingiva, loss of appetite, malaise, malodorous urine, otitis media, painful gingiva/mouth, perioral rash, primary herpetic gingivostomatitis, respiratory problems (including runny nose, congestion, throat infection), restlessness, severe crying, skin rash, sleep disturbance, vomiting, wakefulness, and weight loss [3,5-7]. Aljameel *et al*. [8] also found in a survey that many traditional birth attendants (TBAs) held various beliefs and attitudes toward teething with 97 (59.5%), 91 (55.8 %), and 80 (49.1%) associating teething with fever, diarrhea, and boils respectively. Over two-fifths (44.2%) believed that the teething process caused weight loss in children. How parents can identify typical teething symptoms from other ailments is of serious concern because of potential misdiagnosis and improper home management.

Several studies have concluded that no specific symptoms or cluster of symptoms can reliably predict tooth emergence. Some suggest that symptoms associated with teething are generally not serious, and the presence of fever (>38.5°C) or other clinically important symptoms is unlikely caused by teething [9]. Also, in a study in India, More *et al*. [10] found that parents attributed fever (87%), diarrhea (65%), gum irritation (71%), and the desire to bite (78%) to teething. However, Tsang [3] claims that teething pain remains one of the most sought-after issues in childhood discomfort. Markman [1] suggests that “Teething, like colic, is an ill-defined, non-evidenced-based entity for which parents receive much advice.” It implies that the perception parents and caregivers have about teething influences how they manage it. The management of teething pain and discomfort is both pharmacological and non-pharmacological [3]. Non-pharmacological management includes cooling (chewing chilled teething rings, using cold wet towels, chilled fruits and vegetables like cucumber, carrot, apple, and applying a cold compress to the gums), and rubbing (gingival massage using firm finger pressure, chewing on teething rusks, dry toasts, or pacifiers) [3,5]. Tsang [3] lists paracetamol, ibuprofen, choline salicylates, lignocaine, and benzocaine as pharmacological agents used for the management of teething discomfort. It is commonly observed from many parents that paracetamol, ibuprofen, and teething powder are frequently used for managing teething discomfort. Some also use amoxicillin and metronidazole when the child is passing watery stools (diarrhea). The choices and combinations of drugs for managing teething discomfort vary among parents. The layman's understanding of teething manifestations and management methods has implications for a child´s health. If there is a misdiagnosis and parents use pharmacological components to manage the situation, the drugs´ side effects could be harmful to the child. Moreover, the frequent use of antibiotics such as amoxicillin and metronidazole might result in super infection, resistance, or both. This practice implies that parents could be managing teething discomfort while the actual ailment worsens. Due to the varying views on the presenting symptoms of teething and how teething discomfort is managed by parents across various regions and countries, the researchers deemed it necessary to assess the knowledge and management practices of infant teething symptoms among mothers whose children were admitted to the Pediatric ward of Tamale Teaching Hospital.

## Methods

**Study design:** a prospective descriptive cross-sectional design utilizing a quantitative data collection method was employed to assess the knowledge and management practices of infant teething symptoms among mothers whose children were admitted to the Pediatric ward of Tamale Teaching Hospital.

**Study area:** the research was conducted at Tamale Teaching Hospital (TTH), the largest and only Teaching Hospital in Northern Ghana. Tamale Teaching Hospital is located in Tamale, the regional capital of Northern region. Tamale Teaching Hospital is the largest tertiary health facility (with an 800-bed capacity) in the Northern region, and acts as a referral center for the Savana, the Northeastern, Upper West, and Upper East regions, as well as neighboring Burkina Faso. Ranked as Ghana´s third-largest teaching hospital after the Korle Bu and Komfo Anokye Teaching Hospitals, it offers both general and specialized services. The pediatric ward, where the research was carried out, provides both general and specialist care to patients [11].

**Study population:** the study targeted mothers whose children were admitted to the Tamale Teaching Hospital's Pediatric ward between September 2021 and November 2021. During this period, the ward received a total of 675 pediatric patients.

**Inclusion and exclusion criteria:** all mothers with children admitted to the pediatric ward during the study period were included. However, non-biological caregivers and parents who declined participation were excluded. Additionally, older siblings who brought their younger sick siblings for admission were not considered for the study.

**Sample size and sampling method:** the sample size of 251 was determined using Yamane's [12] formula for finite population, based on the total admissions during the specified period. A convenient sampling strategy was employed to select participating mothers for the study.

**Study variables:** the dependent variable was infant teething (knowledge and management of symptoms). Independent variables included sociodemographic characteristics such as age, marital status, religion, level of education, occupation, hospital ward, number of children, and the age of the youngest child.

**Data collection instrument:** the main instrument for gathering data for the study was a structured questionnaire. The questionnaire was developed in accordance with the study's aims and the existing literature. The questionnaire was used to collect data on; section A): sociodemographic characteristics of mothers; section B): mothers´ knowledge on infant teething; and section C): mothers´ management practices of infant teething symptoms. The survey instrument included only closed-ended questions. Questionnaires were serially numbered to make data input and analysis easier.

**Data collection technique:** following approval from the TTH Research and Development Unit, data collection for this study began. The data collection period lasted from October 2021 to December 2021. After obtaining informed consent, the questionnaires were given to mothers whose children were admitted to the pediatric ward of the hospital. Before administering the questionnaire to them, each questionnaire was coded with an identification number for easy identification. The questionnaires were self-administered, with clarification provided to mothers who needed more information. The completed surveys were cross-checked and sealed in an envelope to ensure that the responses were complete and consistent.

**Validity and reliability:** the questionnaire underwent peer review by pediatric experts to ensure content and face validity. A pretest was conducted among 15 mothers at Tamale West Hospital, using the same inclusion and exclusion criteria to assess accuracy and consistency. No misunderstandings were identified during the pretest; hence, the questionnaire was kept in its original form.

**Data management and analysis:** after field data collection, data were coded, entered, cleaned, and analyzed using Statistical Package for Social Sciences (SPSS) version 21. Descriptive statistics were used to summarize qualitative data. Associations between categorical independent and dependent variables were assessed using a binary logistic regression model, with statistical significance pegged at p < 0.05. Knowledge and practice were classified as good or poor. A total of fourteen (14) items were used to assess mothers' knowledge of infant teething. A median score of 7 was established, where scores below 7 were categorized as “Poor knowledge”, while scores of 7 and above were categorized “Good knowledge”. Similarly, six (6) items were used to measure mothers´ management practices of infant teething symptoms. Using a median score of 3, scores below 3 were categorized as “Poor practice”, and scores of 3 and above were categorized as “Good practice”.

**Ethical consideration:** the study, under the official reference number TTH/R&D/SR/128, obtained ethical approval from the Research and Development Unit of the Tamale Teaching Hospital. Written informed consent was acquired from the mothers before providing them with the questionnaire. They were informed of their right to withdraw from the study at any point without facing any penalties. Anonymity and confidentiality were ensured for all mothers throughout the data collection process.

## Results

**Sociodemographic characteristics of mothers:** a total of 251 mothers, whose children were admitted to the pediatric ward of the Tamale Teaching Hospital (TTH), were involved in the study, resulting in a response rate of 100%. The majority of mothers (47.8%) were aged 30-39 years. Among the mothers, 62.9% were Moslem, 73.3% were married, 35.1% had no formal education, and 51.0% were self-employed. Additionally, 64.9% of the mothers had between 1-3 children, with the youngest children being between 6-12 months (40.6%). This is presented in Table 1.

**Table 1:** sociodemographic characteristics of mothers

Variable	Frequencies (n=251)	Percentages (%)
**Age**		
15-29	106	42.2
30-39	120	47.8
>40	25	10.0
**Marital status**		
Single	43	17.1
Married	184	73.3
Divorced	21	8.4
Widowed	3	1.2
**Religion**		
Christian	93	37.1
Muslim	158	62.9
**Education level**		
Primary	41	16.3
Secondary	49	19.5
Vocational	24	9.6
Tertiary	49	19.6
No formal education	88	35.0
**Occupation**		
Self-employed	128	51.0
Private sector	20	8.0
Government	35	13.9
Unemployed/housewife	68	27.1
**Ward type**		
Pediatric ward	207	82.5
Pediatric emergency ward	44	17.5
**Number of children**		
1-3	163	64.9
4-6	78	31.1
Above 6	10	4.0
**Age of youngest child**		
less than 6 months	59	23.5
6-12 months	102	40.6
13-24 months	59	23.5
above 24 months	31	12.4

**Mothers´ knowledge on infant teething:** as presented in Table 2, the majority of mothers had an idea of what teething was. Specifically, 59.8% described teething as the permanent eruption of teeth in children, 52.6% indicated babies´ teeth start to erupt around 6-7 months of age, 58.6% indicated the first teeth that appear in the mouth are lower central incisors, and 53.0% indicated the eruption of teeth does not complete at approximately 2 years of age. Also, the majority of respondents (66.6%) responded that delayed eruption of teeth may not be an indication of systemic disease. Regarding teething symptoms, the majority of mothers (84.1%) identified fever, sleep disturbance (60.2%), gum irritation (54.2%), desire to bite (66.5%), increased salivation/drooling (60.2%), and poor feeding (65.3%). The study indicated that 79.7% of mothers had good knowledge of teething, while 20.3% had poor knowledge.

**Table 2:** mothers’ knowledge of infant teething

Variable	Subgroup	Frequency (n=251)	Percentage
General knowledge on infant teething			
Teething describes permanent tooth eruption in children	Yes	150	59.8
	No	101	40.2
Babies’ teeth start to erupt around 6-7 months of age	Yes	132	52.6
	No	119	47.4
The first teeth to appear in the mouth are lower central incisors	Yes	147	58.6
	No	104	41.4
The eruption of teeth gets completed at approximately 2 years of age	Yes	118	47.0
	No	133	53.0
Delayed eruption of teeth may be an indication of the presence of systemic disease	Yes	84	33.5
	No	167	66.6
Do you worry about the time your baby’s teeth start to erupt?	Yes	153	61.0
	No	98	39.1
Do you think babies have any problems when their teeth are erupting?	Yes	179	71.3
	No	72	28.7
**Knowledge of symptoms of infant teething**			
Fever	Yes	211	84.1
	No	40	15.9
Sleep disturbance	Yes	151	60.2
	No	100	39.8
Gum irritation	Yes	136	54.2
	No	115	45.8
Gum swelling	Yes	107	42.6
	No	144	57.4
Desire to bite	Yes	167	66.5
	No	84	33.5
Increased salivation/drooling	Yes	151	60.2
	No	100	39.8
Poor feeding	Yes	164	65.3
	No	87	34.7

**Mothers´ management practices of infant teething symptoms:** in Table 3 shows that a majority of mothers (76.1%) took the child to the hospital during teething symptoms as a standard practice. Nearly all mothers (90.8%) administered Paracetamol syrup/suppository to relieve symptoms, and 82.1% used 'Teedar´ syrup. Moreover, 81.7% consulted a physician about teething symptoms. Again, 34.7% used a pacifier to alleviate children´s symptoms, and 47.4% used teething powder. The study indicated that 65.3% of the mothers had good practices, while 34.7% had poor practices in managing teething symptoms.

**Table 3:** mothers’ management practices of infant teething symptoms

Variable	Subgroup	Frequency (n=251)	Percentage
Take the child to the hospital during teething	Yes	191	76.1
	No	60	23.9
Give Paracetamol syrup/suppository	Yes	228	90.8
	No	23	9.2
Give pacifier	Yes	87	34.7
	No	164	65.3
Give teething powder	Yes	119	47.4
	No	132	52.6
Give ‘Teedar’ syrup	Yes	206	82.1
	No	45	17.9
Consult a physician	Yes	205	81.7
	No	46	18.3

**Factors associated with mothers´ knowledge of infant teething:** logistic regression analysis demonstrated significant associations between marital status (p= 0.029) and type of ward (p= 0.020) with mothers' knowledge of teething. This is shown in Table 4.

**Table 4:** factors associated with mothers’ knowledge of infant teething

Variable	B	SE	P-value	Odds ratio
Age	-0.013	0.241	0.958	0.98
Marital status	-0.660	0.303	0.029*	0.52
Religion	-0.076	0.321	0.813	0.92
Educational level	0.181	0.107	0.092	1.20
Occupation	0.058	0.118	0.626	1.06
Ward type	-1.444	0.620	0.020*	0.24

* *Statistically significant p < 0.05, B: Unstandardized beta coefficient, SE: standard error

**Predictors of mothers´ management practices of infant teething symptoms:** the logistic regression analysis identified significant associations between mothers´ age, educational level, and the type of ward with the management practices of teething symptoms. In the multivariate model, the results showed that mothers less than 30 years were 2.07 times more likely to have good management practices of teething symptoms (OR, 2.07; 95% CI: 1.19-3.57; p= 0.009) compared to mothers above 30 years. Similarly, mothers with formal education were 2.22 times more likely to have good management practices of teething symptoms (OR, 2.22; 95% CI: 1.22-3.81; p= 0.004) compared to those without formal education. Furthermore, mothers in the pediatric ward “W” were 4.40 times more likely to have good management practices of teething symptoms (OR: 4.40; 95% CI: 2.21-8.70; p= 0.000) compared to those in pediatric ward “E”. This is presented in Table 5.

**Table 5:** predictors of mothers’ management practices of infant teething symptoms

Variable	Sub-group	Mother’s management practices of teething symptoms		Total (Percentages)	P-value	COR/95% CI
		Good	Poor			
Age	<30	27	79	106 (25.5) (74.5)	0.009*	2.07 (1.19-3.57)
	>30	26	75	145 (41.4) (58.6)		
Religion	Christianity	26	67	93 (28.0) (72.0)	0.088	0.62 (0.35-1.08)
	Islam	61	97	158 (38.6) (61.4)		
Education level	Formal education	46	117	163 (28.2) (71.8)	0.004*	2.22 (1.22-3.81)
	No formal education	41	47	88 (46.6) (53.4)		
Marital status	Currently married	63	121	184 (34.2) (65.8)	0.951	1.03 (0.51-2.07)
	Ever been married	9	15	24 (37.5) (62.5)	0.936	0.89 (0.32-2.52)
	Single	15	28	43 (34.9) (65.1)	0.830	
Occupation	Employed	62	121	183 (33.9) (66.1)	0.670	1.14 (0.64-2.03)
	Unemployed	25	43	68 (36.8) (63.2)		
Number of children	1-3	58	105	163 (35.6) (64.4)	0.676	0.90 (0.55-1.54)
	>3	29	59	88 (33.0) (67.0)		
Ward type	Pediatric W	59	148	207 (28.5) (71.5)	0.000*	4.40 (2.21-8.70)
	Pediatric E	28	16	44 (63.6) (36.4)		
Knowledge level	Good	65	134	199 (32.7) (67.3)	0.195	1.51 (0.81-2.82)
	Poor	22	30	52 (42.3) (57.7)		

* Statistically significant p<0.05, COR, Crude Odds Ratio, CI: Confidence interval Note: Self-employed, private sector, government were grouped as employed whiles unemployed/house wife were considered as employed. primary, secondary, vocational and tertiary were considered as formal education versus no-formal education. Divorced and widowed were considered as ever been married, married as currently married and single remains single.

## Discussion

The study sought to assess the knowledge and management practices regarding infant teething symptoms among mothers whose children were admitted to the pediatric ward of Tamale Teaching Hospital. All mothers demonstrated an understanding of infant teething. The majority of them defined teething as the permanent eruption of teeth in children. A greater proportion (59.8%) mentioned the initiation of teething between 6 and 7 months of age, while 58.6% of respondents believed the first teeth that appear in the mouth are lower central incisors. This knowledge may be attributed to health education given at the Child Welfare Clinic (CWC) in the study area and the educational level of respondents. These findings resonate with the study findings in Saudi Arabia [2] and South India [13], which reported that a greater part of the study respondents said that the first teeth emerged between 6-7 months and the first teeth to erupt are the central lower incisors. A majority of mothers (53.0%) did not believe the eruption of teeth complete at approximately 2 years of age. This finding could be related to the observations of mothers on their children. However, this is in contrast with previous studies which report that mothers indicated that the eruption of teeth is completed by age two [14,15]. The majority of mothers (66.6%) did not believe delayed eruption of teeth may be an indication of systemic disease. This, however, does not resonate with the findings in Saudi Arabia [2], which indicated that delay in tooth eruption is a result of the presence of a disease. The inconsistency could be related to the difference in race and setting.

Regarding the mothers´ knowledge of teething symptoms, the majority (84.1%) identified fever as a symptom, with sleep disturbance (60.2%), gum irritation (54.2%), desire to bite (66.5%), increased salivation/drooling (60.2%), poor feeding (65.3%), and almost half (42.6%) mentioning gum swelling as a symptom of teething. These findings could be due to the experience of mothers during their children´s teething process. This is consistent with research findings in Nigeria [6] and Saudi Arabia [2], which reported that a great number of mothers attributed teething with fever, sleeplessness, gum irritation, and increased salivation. However, the findings of this study do not collaborate with the report in Nigeria [16], which stated signs and symptoms of teething as ear infection and cough. This inconsistency could be attributed to differences in the experiences of mothers regarding infant teething. On assessing the management practices of mothers regarding teething symptoms, this study showed that the majority acknowledged taking the child to the hospital as standard practice. A total of 90.8% of respondents gave paracetamol syrup/suppository to relieve the symptoms, while 82.1% administered ‘Teedar’ Syrup. A greater proportion of mothers (81.7%) consulted a physician concerning the teething symptoms. However, about a third of the mothers (34.7%) relieved their children´s symptoms by giving a pacifier, and almost half (47.4%) used teething powder. These findings are consistent with previous studies from Nigeria [16], Mysore [15], Nigeria [6], and Saudi Arabia [17], which reported that the majority of mothers preferred consulting physicians, using pacifiers, and administering pain relievers. This is also similar to the research on teething symptoms and management during infancy from South Africa [18] and Ethiopia [14], which reported that mothers mostly consulted health personnel during their children´s teething processes. These similarities could be attributed to intense health education at health facilities and advertisements of medication on social media relating to teething in children. However, this study does not resonate with the findings from India [10], which reported the use of Tlisma necklace and home medication by some parents.

This inconsistency could be related to the different settings of the study. In assessing the predictors of mothers´ management practices of teething symptoms, it was found that mothers who had formal education were 2.22 times more likely to have good management practices of teething symptoms (AOR, 2.22; 95% CI: 1.22-3.81; p= 0.004) than those with no formal education. Formal education provides a mother with access to information about child development, including teething. By understanding the process of teething and its associated symptoms, a mother can better recognize and differentiate them from other potential health issues. This study conforms with the study from Nigeria [6], which reported that mothers with low levels of education have a high risk of recommending or giving concoctions and antibiotics during teething processes in children. This is similar to the report from Mysore [15], which reported that the educational level of respondents is significantly associated with the myths surrounding teething challenges. These similarities could be due to the level of exposure of mothers to knowledge of health matters.

**Limitations:** the findings of this research should be interpreted while considering certain limitations. Firstly, the study adopted a cross-sectional design and relied on self-reported evaluations, which increases the likelihood of underreporting. Additionally, it is important to note that the study was conducted exclusively in a single hospital within the Northern Region of Ghana, making it inappropriate to generalize these results to the entire region.

## Conclusion

According to the findings of the current study, most mothers have a good understanding of child teething, but they do not think delayed eruptions indicate systemic disease. They identified taking the child to the hospital during teething symptoms and administering paracetamol to relieve the symptoms as standard practices. However, more education is needed to differentiate between teething signs and other ailments and to prevent substandard first aid practices during teething.

### What is known about this topic

- *Teething is a natural process in infants where most toddlers get their first tooth around six months of age and a full set of 20 deciduous teeth by 30 months;*
- *Parental perceptions of teething symptoms vary across different backgrounds, education levels, genders, and regions;*
- *Symptoms associated with teething include fever, sleep disturbance, gum irritation, increased salivation, desire to bite, poor feeding, and gum swelling*.

### What this study adds

- *Mothers with formal education are more likely to have better practices in managing teething symptoms compared to those without formal education*;
- *Cultural beliefs and regional disparities influence the management of teething symptoms*;
- *Consulting healthcare professionals and using pain relievers are common management practices of teething symptoms among mothers*.
